# Impact of Laser Speed and Drug Particle Size on Selective Laser Sintering 3D Printing of Amorphous Solid Dispersions

**DOI:** 10.3390/pharmaceutics13081149

**Published:** 2021-07-27

**Authors:** Rishi Thakkar, Miguel O. Jara, Steve Swinnea, Amit R. Pillai, Mohammed Maniruzzaman

**Affiliations:** 1 Pharmaceutical Engineering and 3D Printing (PharmE3D) Lab, Division of Molecular Pharmaceutics and Drug Delivery, College of Pharmacy, The University of Texas at Austin, Austin, TX 78712, USA; rishithakkar@utexas.edu (R.T.); arpillai@utexas.edu (A.R.P.); 2 Division of Molecular Pharmaceutics and Drug Delivery, College of Pharmacy, The University of Texas at Austin, Austin, TX 78712, USA; miguel.jara@utexas.edu; 3 Department of Chemical Engineering, Texas Materials Institute, The University of Texas at Austin, Austin, TX 78712, USA; swinnea@che.utexas.edu

**Keywords:** selective laser sintering, 3D printing, amorphous solid dispersion, solubility enhancement, residual crystallinity, laser speed, drug particle size

## Abstract

This research demonstrates the influence of laser speed and the drug particle size on the manufacturing of amorphous solid dispersions (ASD) and dosage forms thereof using selective laser sintering 3-dimensional (3D) printing. One-step manufacturing of ASD is possible using selective laser sintering 3D printing processes, however, the mechanism of ASD formation by this process is not completely understood and it requires further investigation. We hypothesize that the mechanism of ASD formation is the diffusion and dissolution of the drug in the polymeric carrier during the selective laser sintering (SLS) process and the drug particle size plays a critical role in the formation of said ASDs as there is no mixing involved in the sintering process. Herein, indomethacin was used as a model drug and introduced into the feedstock (Kollidon^®^ VA64 and Candurin^®^ blend) as either unprocessed drug crystals (particle size > 50 µm) or processed hot-melt extruded granules (DosePlus) with reduced drug particle size (<5 µm). These feedstocks were processed at 50, 75, and 100 mm/s scan speed using SLS 3D printing process. Characterization and performance testing were conducted on these tablets which revealed the amorphous conversion of the drug. Both MANOVA and ANOVA analyses depicted that the laser speed and drug particle size significantly impact the drug’s apparent solubility and drug release. This significant difference in performance between formulations is attributed to the difference in the extent of dissolution of the drug in the polymeric matrix, leading to residual crystallinity, which is detrimental to ASD’s performance. These results demonstrate the influence of drug particle size on solid-state and performance of 3D printed solid dispersions, and, hence, provide a better understanding of the mechanism and limitations of SLS 3D printing of ASDs and its dosage forms.

## 1. Introduction

Selective laser sintering (SLS) is a powder bed-based 3-dimensional (3D) printing platform which has gained interest amongst pharmaceutical researchers in the last decade [1]. 3D printing processes can be classified based on the state of feed material they use (feedstock) [2]. Material extrusion and material jetting processes utilize filaments [3], and semi-solids or melted material [4,5], respectively. Whereas powder bed fusion and binder jetting utilize powders as feedstock [6,7], and the point of difference is the basic additive manufacturing principle where the former is based on the selective fusion of material in the powder bed [8], and the latter is based on reactive curing of the material in the powder bed [9]. These processes and feedstock utilize thermal reaction bonding except for binder jetting which, despite being a powder bed-based platform, utilizes the basic AM principle of chemical reaction bonding, which is similar to the AM processes such as material jetting, and vat photopolymerization [10,11]. The SLS 3D printing process falls under powder bed fusion processes which involves a powder bed-based feedstock layer being selectively fused utilizing a thermal stimulus. SLS processes, unlike selective laser melting (SLM), do not involve complete melting and solidification of the material, but the fusion of particle surfaces during the printing process [12].

SLS 3D printing can be further divided based on the laser source (e.g., visible, CO_2_, and Near Infrared) and the feedstock material (polymeric, metallic, ceramic, and composite). For pharmaceutical applications, the process utilizes thermoplasticity of the feedstock components due to the presence of pharmaceutical polymers. Research in this area has demonstrated the application of SLS in 3D printing of modified-release oral solid dosage forms including orally disintegrating [13], immediate-release [14,15], and sustained release [16,17] tablets. Moreover, the versatility induced by AM has allowed the printing of multi-material [18], multi-drug printlets [19], multi-layered lattices [20], medical implants [21,22,23], and devices [24]. This previous and currently ongoing research depicts the potential of SLS 3D printing in manufacturing a wide range of robust pharmaceutical dosage forms with different functionalities.

In our previous research, we demonstrated the use of SLS as a one-step manufacturing platform for amorphous solid dispersions using ritonavir as a model drug [25]. Over 90% of the drugs in the new chemical entity (NCE) pipeline and 36% of the drugs in the market are either biopharmaceutical class (BCS) II or IV, which consists of drugs with poor water solubility [26,27]. Amorphous solid dispersions (ASD) are amongst different supersaturating formulation strategies developed to attain, stabilize and maintain the drug’s kinetic solubility, thereby observing a significant solubility enhancement and in vivo bioavailability of the dosage forms [28,29]. ASD are manufactured using hot-melt extrusion (HME), co-precipitation, and spray drying (SD) on a commercial scale [30,31]. However, the development of novel strategies for ASD manufacturing has been an active area of research with emerging and promising platforms such as KinetiSol^®^ under development [32,33,34,35,36,37]. Our previous research shows that knowledge of formulation properties including thermal properties of the components and solubilization capacity [38] of the polymer along with a suitable combination of processing parameters, i.e., surface temperature, hatching spacing, and laser speed can be used for 3D printing amorphous solid dispersions [25,39]. In the previous study, the SLS printed ritonavir tablets demonstrated a 21-fold increase in solubility in vitro [25]. One critical formulation aspect involved was the flow properties of the formulation (feedstock powder blend), which interfered with the SLS 3D printing process and led to inconsistencies in the quality of the printed dosage forms. To circumvent this problem we developed a hot melt extrusion-based platform for the continuous manufacturing of granules with excellent uniformity and flow properties suitable for SLS 3D printing and used this platform for manufacturing ASDs of indomethacin [40].

This present research compares indomethacin granules and tablets manufactured using DosePlus technology to the unprocessed drug crystals by means of SLS 3D printing. We hypothesize that the mechanism of ASD formation in the absence of mixing is the diffusion of the drug in the molten polymer and further dissolution of the drug in the matrix forming a solid dispersion. If this is the case, the particle size of the drug in the formulation will have a significant effect on the ASD formation, where a lower particle size will diffuse and dissolve faster whereas a larger particle size will take time. We have further inspected the difference in the solid-state and statistically compared the performance of these two formulations.

## 2. Material and Methods Materials

### 2.1. Materials

HPLC grade acetonitrile was purchased from Fisher Scientific (Pittsburg, PA, USA); all other chemicals and reagents were ACS grade or higher. Indomethacin (Tokyo Chemical Industries, Lot no. D3NIJJR, Tokyo, Japan), vinyl pyrrolidone-vinyl acetate copolymer (Kollidon^®^ VA 64 (average molecular weight 65,000 g/mol), Lot no. 94189624U0, BASF, Ludwigshafen, Rhineland-Palatinate, Germany), silicon dioxide (FujisilTM, Lot no. 906003, Fuji chemical industries co., Ltd. Toyama pref., Toyama, Japan), polysorbate 80 (Lot no. BCCB4768, Sigma-Aldrich^®^, St. Louis, MO, USA), magnesium aluminometasilicate (Neusilin US 2, Lot no. 804011, Fuji chemical industries co., Ltd. Toyama pref., Toyama, Japan), potassium aluminum silicate-based pearlescent pigment (Candurin^®^, Lot no. W150645X08, Merck KGaA, Darmstadt, Hesse, Germany).

### 2.2. Feedstock Preparation

One aspect of this study was to investigate the influence of drug particle size resulting from different ways of feedstock preparation and its influence on the formation of ASD using selective laser sintering. The unprocessed feedstock for SLS 3D printing was prepared by physically blending the drug (40% *w*/*w*) with the inorganic carriers, colloidal silicon dioxide (27.5% *w*/*w*), magnesium aluminometasilicate (27.5% *w*/*w*) using a high shear mixer (Robot Coupe, USA Inc., Ridgeland, MS, USA) and polysorbate 80 (5% *w*/*w*) was added to the mixer dropwise. This prepared blend with the crystalline drug (25% *w*/*w*) was again blended with 3% *w*/*w* Candurin^®^ and 72% *w*/*w* Kollidon^®^ VA 64 to prepare the final feedstock for SLS 3D printing. This feedstock with the crystalline unprocessed drug will now be referred to as ‘unprocessed feedstock (UFS)’. The manufacturing and characterization of the processed granules have been reported in our previous work [40]. In a nutshell, the physical blend containing 40% *w*/*w* drug, 27.5% *w*/*w* colloidal silicon dioxide, 27.5% *w*/*w* magnesium aluminometasilicate, and 5% *w*/*w* polysorbate 80 were blended using a high shear mixer. This blend was gravimetrically (twin-screw gravimetric feeder, Brabender Technologie, Ontario, Canada) fed to a hot-melt extruder (ZSE 12 HP-PH, Leistritz Advanced Technologies Corp., Nuremberg, Germany) with a 12 mm outer diameter (OD) at a feeding rate of 5 g/min. The granules were manufactured at a 155 °C and the screw speed was set to 30 RPM. These collected granules were blended with 3% *w*/*w* Candurin^®^ and 72% Kollidon^®^ VA 64 for SLS 3D printing. This feedstock with processed granules will be referred to as ‘processed feedstock (PFS)’. The UFS and PFS prepared were used as the feedstock for the SLS 3D printing process. This prepared feedstock was passed through a number 140 sieve (105 µm). The particle size distributions (PSDs) of both UFS and PFS were measured using a RODOS disperser coupled to a Sympatec laser diffractor unit (Sympatec, Clausthal-Zellerfeld, Germany) at 3.0 bar and 20% feed table rotation. Primary particle size distribution (PPSD) distribution (Figure S1 and Table S1) for the PFS was found to be *d*_10_ = 2.28 µm, *d*_50_ = 50.99 µm, *d*_90_ = 105.47 µm, whereas that for the UFS was found to be *d*_10_ = 2.63 µm, *d*_50_ = 46.15 µm, *d*_90_ = 100.96 µm which are acceptable for SLS 3D printing. However, it should be noted that these values represent the particle size distribution of the feedstock with all the components blended and not the drug’s particles size alone. The particle size of the drug in UFS and PFS is depicted in the results section using microscopic analysis like SEM analysis.

### 2.3. Differential Scanning Calorimetry (DSC)

DSC was used to investigate the crystallinity and degradation of the drug during the granulation or SLS 3D printing process and the thermal behavior of the formulation components which aids in the selection of SLS 3D printing parameters. The pure drug, polymer, UFS, PFS, and SLS 3D printed tablets manufactured at different laser speeds (50, 75, and 100 mm/s) were analyzed using a benchtop DSC (DSC Q20, TA^®^ instruments, New Castle, DE, USA). All the samples were weighed (5–15 mg) using a microbalance (Sartorius 3.6P microbalance, Göttingen, Germany) and transferred to tared standard aluminum pans (DSC consumables incorporated, Austin, MN, USA) and were then sealed using standard aluminum lids. The start and stop temperatures were 30 °C and 200 °C respectively, and the ramp rate used was 10 °C/min.

### 2.4. Selective Laser Sintering (SLS)

The prepared feedstock was added to the reservoir chamber of the SLS 3D printer (Sintratec kit, Sintratec, Brugg, Switzerland). For each printing batch, 100 g of feedstock was used. A tablet with 6.5 mm height and 13 mm diameter was designed using 3D builder software (Microsoft Corporation, Washington, DC, USA), however, tablets with different dimensions were designed and printed for the dissolution test to attain a similar dose of 30 mg under different laser speeds using the volume and density calculations as per Equations (1) and (2). The ‘.STL’ file was opened in the Sintratec central software (version 1.2.0), 12 tablets were arranged in the print chamber using the slicing software (Sintratec Central). The layer height, number of perimeters, and perimeter offset were set to 100 µm, 1, and 200 µm, respectively. The Hatching offset and the hatch spacing were set to 120 µm, and 25 µm, respectively. During the printing process, the chamber was maintained at 90 °C and the surface temperature was set to 100 °C. Both these processing temperatures were set slightly below the glass transition point of Kollidon^®^ VA64 (>120 °C) and significantly below the drug’s melting point (153 °C). The laser speed was varied between the three processed batches of the UFS and the PFS at 50 mm/s, 75 mm/s, and 100 mm/s, and all the other print parameters and processing parameters were kept constant. After each batch was printed the tablets were collected and dedusted using an air gun to remove the loosely bound powder on the prints. After de-dusting, the weight and dimensions of tablets from each batch were measured using a calibrated balance, and vernier caliper, respectively. Three tablets from each batch were also tested for hardness using a texture analyzer (TA-XT2 analyzer, Texture Technologies Corp, New York, NY, USA). The rest of the tablets were used to evaluate the disintegration time (*n* = 3), and other solid-state characterizations including digital microscopy (Dino light, Torrance, CA, USA), X-ray diffraction, differential scanning calorimetry, and dynamic vapor sorption analysis.

(1) $$Volume\;\left(V\right)=\pi r^{2}h$$

(2) $$Density\;\left(\rho \right)=\frac{mass}{volume}$$

### 2.5. Scanning Electron Microscopy (SEM)

The particle size of the drug in the sieved PFS and the UFS was determined using a scanning electron microscope (Quanta FEG 650 ESEM, FEI Company, Hillsboro, OR, USA). The PFS and UFS were dispensed onto a carbon tape and gold-sputtered (EMS Sputter Coater, Hatfield, PA, USA). The samples were then loaded onto the SEM sample stage and images were captured using a 10 kV accelerated voltage, and 15 µÅ emission current. The working distance for the analysis was maintained at 10 mm, and the spot size was set to 3. To demonstrate the particle size of the drug, the images for the two feedstocks were taken of varying magnifications i.e., 400× for the UFS and 2000× for the PFS. Images of the samples at comparative magnifications can be found in our previously published work [40].

### 2.6. Hot Stage Microscopy (HSM)

Hot stage microscopy was used to mimic the conditions the feedstock experiences during the SLS 3D printing process. A thin layer of the sieved UFS and PFS were carefully spread on a glass slide and the glass slide was covered with a coverslip. The sample slide was placed on the microscope stage of an Olympus BX53 polarized photomicroscope (Olympus America Inc., Webster, TX, USA) equipped with Bertrand Lens. The sample zone was selected using a 10× lens and after focusing on the sample the lens was shifted to 20× magnification. The UFS and PFS were exposed to a starting temperature of 90 °C and the temperature was ramped up to 150 °C, which was then ramped down to 90 °C after holding it at 150 °C for a minute. Further, to assess the crystallinity and morphology of printed tablets, samples processed at different laser speeds were observed for birefringence with and without a 530 nm compensator (U-TP530, Olympus^®^ Corporation, Shinjuku City, Tokyo, Japan) using a QICAM Fast 1394 digital camera (QImaging, Surrey, BC, Canada).

### 2.7. Powder X-ray Diffraction (PXRD)

The crystallinity of the drug was determined using a benchtop PXRD equipment (MiniFlex, Rigaku Corporation, Tokyo, Japan). 50–100 mg of the pure drug, polymer, UFS, PFS, and SLS 3D printed tablets manufactured at different laser speeds (50, 75, and 100 mm/s) were dispensed onto the sample cell. A glass slide was used to flatten the surface and remove the excess powder before placing these cells on the sample holder. The samples were analyzed at a 2θ angle from 5 to 45 degrees and a scan speed of 2 degrees/min. The scan step was set to 0.02 degrees and the scan resolution was 0.0025 for the analysis at a 45 V voltage and the 15-mV current, respectively.

### 2.8. Dynamic Vapor Absorption (DVS)

DVS analysis was performed on the 3D printed tablets manufactured with UFS and PFS at different laser speeds (50 mm/s, 75 mm/s, and 100 mm/s) to understand the behavior and stability of different formulations using a DVS Resolution gravimetric sorption equipment (Surface measurements Ltd., Allentown, PA, USA). About 7–15 mg of the crushed tablets were loaded in the sample pan after it was tared and zeroed. Once the sample cell weight stabilized (dm/dt was <0.002%/min), a full cycle of the DVS was run from 0% RH to 100% RH with a 20% RH interval at 25 °C. The carrier gas and solvent used for the analysis were nitrogen and water. The step-change was controlled using dm/dt (<0.002%/min) not time to make sure that the %RH changes only once the weight change was stable i.e., once absorption/desorption is complete. The analysis was controlled using DVS control software (Surface measurements Ltd., Allentown, PA, USA).

### 2.9. In Vitro Drug Release Performance Testing

In our previous study, we conducted the dissolution test in sink conditions to evaluate the performance of 3D printed ASD. To assess the solubility advantage of the 3D printed ASDs we conducted a non-sink pH shift (pH 2 to pH 6.8) in vitro dissolution test using a standard dissolution apparatus with 150 mL dissolution vessels and paddles (Vankel VK 7000, Agilent Technologies, Santa Clara, CA, USA) at 37.5 °C [41]. The tablets were exposed to 100 mL of HCl-KCl buffer (pH 2) for 1 h and then diluted with 50 mL of concentrated phosphate buffer to pH 6.8 for 3 h, with paddles at 50RPM. The samples were withdrawn from the vessel using 0.2 µm polyethersulfone filters (VWR International, Radnor, PA, USA) at predetermined time points, diluted two folds with acetonitrile, and analyzed using reverse-phase high-performance liquid chromatography (HPLC) as per previously calibrated protocol [40]. The equilibrium solubility of indomethacin over 24-h was found to be 24.6 µg/mL at pH 6.8. Using the solubility of indomethacin (C_s_), the dissolution vessel volume (V), and the minimum drug content of indomethacin (Dose) in the tablets the sink index was found to be 0.12 using Equation (3) which is considered as perfect sink conditions as per previously conducted research [42]. Furthermore, to statistically assess the impact of laser speed (50 mm/s, 75 mm/s, and 100 mm/s), drug particle size (UFS and PFS), and a combination of the two factors, a full factorial, repeated measures MANOVA analysis was conducted to compare the release and solubility profiles of the different formulations. Furthermore, a one-way ANOVA between the three laser speed groups (50 mm/s, 75 mm/s, and 100 mm/s) and a pooled ‘t’ test between the two processing groups (UFS and PFS) were also conducted. These statistical tests can determine whether the influence of these factors is significant or not between and within groups, however, they fail to demonstrate the source of variability i.e., which formulations are significantly different. To determine whether the formulations were significantly different MANOVA analysis was conducted for the solubility and release from different formulation over time, and ANOVA was conducted for the formulations at T = 240 min to assess which formulations are significantly from each other. Here each formulation is defined as the combination of laser speed (50 mm/s, 75 mm/s and 100 mm/s) and processing group (UFS and PFS), there resulting in six unique formulations, where each is considered as one group for the statistical test. The statistical analysis was conducted using JMP^®^ software (JMP^®^ 15.0.0, SAS Institute Inc., Cary, NC, USA).

(3) $$SI=\frac{Cs\times V}{Dose}$$

### 2.10. Wide-Angle X-ray Scattering (WAXS)

The wide-angle X-Ray Scatterings (WAXS) technique has been previously used to evaluate the trace crystallinity within the printed tablets [25] and other solid samples, however in this study it was used to determine the solid-state of the suspended particles in the dissolution medium. Samples were withdrawn from the dissolution vessels of UFS and PFS tablets sintered at 100 mm/s after 1 h in pH 2 and 3 h in pH 6.8. The samples were added to 1 mm glass capillaries (Hampton Research, Aliso Viejo, CA, USA) using a 24 G, 80 mm syringe and analyzed using A SAXSLab instrument (WAXSLab, Northampton, MA, USA). The PILATUS3 R 300 K detector (DECTRIS Ltd., Philadelphia, PA, USA) equipped contains 3 detecting modules with an area of 83.8 × 106.6 mm^2^ and a pixel size of 172 × 172 µm^2^. This instrument was equipped with a microfocus Cu K-alpha rotating anode X-ray source which was operated at 50 kV and 0.6 mA. The instrument was controlled, and measurement was set using Ganesha instrument software (SAXSLab, Northampton, MA, USA) where the distance between the detector and sample ranged from 0.95 to 1.45 m. Each sample was analyzed for an acquisition time of 600 s and a 2 mm off-centered beam stop mask. SAXSGUi software (SAXSLab, Northampton, MA, USA) was used to analyze the collected data.

### 2.11. Particle Size Analysis

Samples were withdrawn from the dissolution vessel after exposing the tablets to 1 h of pH 2 and 4 h of pH 6.8. These withdrawn samples were then filtered using 0.2 µm nylon syringe filters (VWR International, West Chester, PA, USA). The particle size of the components in these filtered samples was measured using a Zetasizer Nano ZS (Malvern Instruments Ltd., Worcestershire, UK) and the Dip cell ZEN1002. Water was selected as the dispersant, and the samples were measured using the 173 backscatter with automatic measurement duration.

## 3. Results

### 3.1. SEM and HSM Analysis

**Figure 1:**
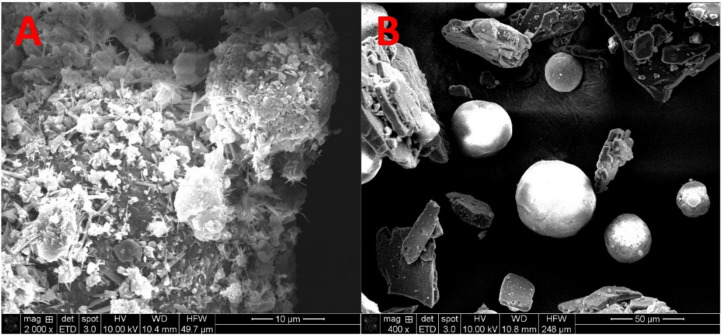
Scanning Electron Microscopy demonstrating the particle size of the drug in (**A**) PFS and (**B**) UFS.

The key difference between PFS and UFS is that in the PFS the drug is absorbed onto inorganic carriers and the particle size of the absorbed drug is less than 5 µm, whereas the particle size of drug crystals in the UFS is greater than 50 µm as seen in Figure 1. The SEM images demonstrate the significant reduction in the drug’s particle size post HME processing. This reduction in particle size and the drug’s absorption on the inorganic carrier not only improves the feedstock flow properties but also improves the drug content uniformity in the feedstock as demonstrated in the previous study [40]. In this study, we have attempted to understand the influence of this reduced particle size on the ASD formation during the SLS 3D printing. Theoretically, the mechanism of ASD formation by SLS 3D printing is the diffusion and dissolution of the drug in the molten polymer when the powder is exposed to the laser. In this case, the reduced particle size should aid this mass transfer phenomenon (diffusion and dissolution) because of an increase in the total surface area.

**Figure 2:**
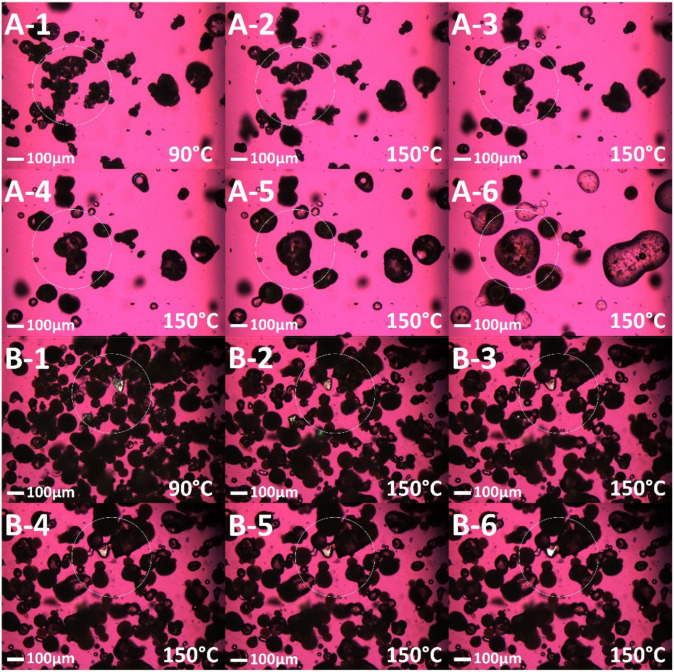
Hot stage microscopy images of PFS (**A-1**–**A-6**) and UFS (**B-1**–**B-6**) at different temperatures.

**Figure 3:**
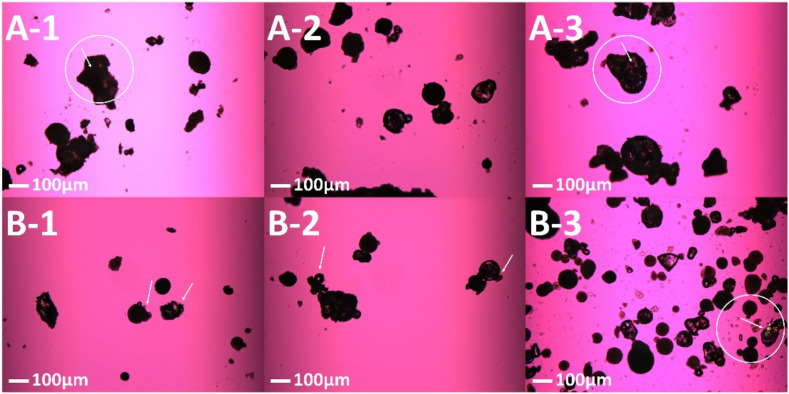
Polarized light microscopy of SLS 3D printed tablets from PFS and UFS (**A-1**) 50 mm/s PFS, (**A-2**) 75 mm/s PFS, (**A-3**) 100 mm/s PFS, (**B-1**) 50 mm/s UFS, (**B-2**) 75 mm/s UFS, (**B-3**) 100 mm/s UFS.

**Figure 4:**
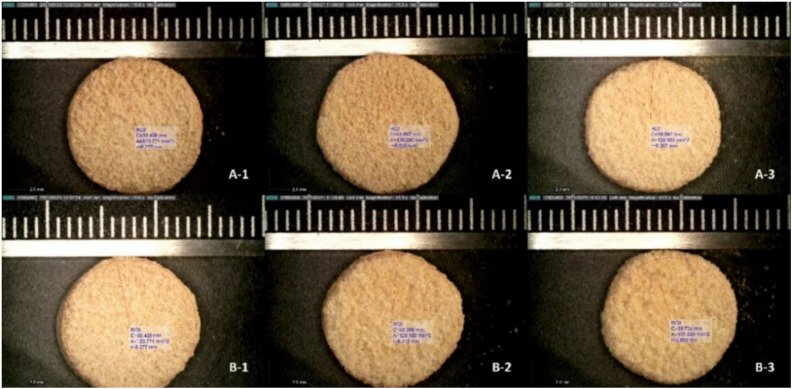
Digital microscopy of SLS 3D printed tablets from PFS and UFS (**A-1**) 50 mm/s PFS, (**A-2**) 75 mm/s PFS, (**A-3**) 100 mm/s PFS, (**B-1**) 50 mm/s UFS, (**B-2**) 75 mm/s UFS, (**B-3**) 100 mm/s UFS. (Each bar is 1 mm).

**Figure 5:**
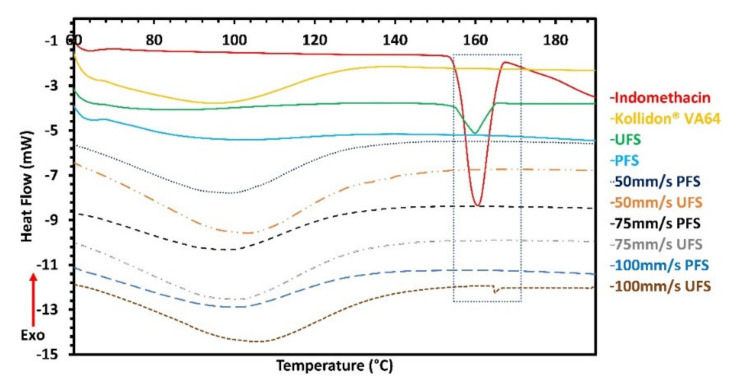
Differential scanning calorimetry of pure drug, polymer, feedstocks, and 3D printed tablets.

**Figure 6:**
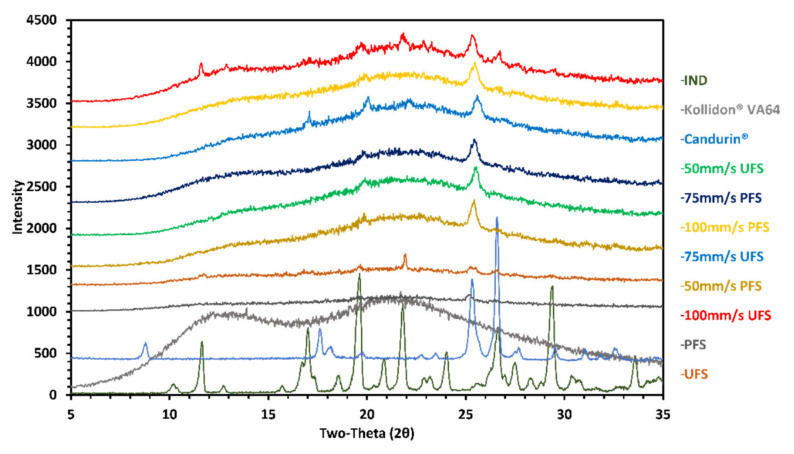
X-ray diffraction of pure drug, polymer, feedstocks, and 3D printed tablets.

In this study, HSM was used to elucidate the mechanism of ASD formation during the SLS 3D printing process and the influence of the drug’s particle size on this process. Since the chamber temperature is maintained at 90 °C during the SLS 3D printing process, the glass sample slide was exposed to 90 °C for the initial 5 min. Figure 2(A-1,B-1) represents the state of the powder feedstock after being exposed to 90 °C for 5 min. After this initial period, the temperature was ramped up to 150 °C, which is above the glass transition temperature of the polymer (≈120 °C) but below the melting temperature of the drug (>153 °C). The transition of the polymer and the cascade of events following this increase in temperature have been demonstrated in Figure 2 for both the PFS and UFS. For the PFS the round dark particles are the processed granules, and the glassy irregular particle is the polymer. The polymer starts transiting in Figure 2(A-2) and engulfs the neighboring granule in Figure 2(A-3) which is followed by the dissolution of the drug in the polymeric matrix as seen in Figure 2(A-4–A-6). This phenomenon has been demarked by a white circle; however, similar occurrences can be seen throughout the zone of focus in the figures. The phenomenon occurring in UFS is shown in Figure 2, where Figure 2(B-1) depicts the state of the feedstock after 5 min at 90 °C. The drug crystals are surrounded by the inorganic carrier and the polymer and after ramping up the temperature at 150 °C the surrounding polymer starts melting which can be seen in the subsequent figures. In Figure 2(B-5,B-6) the drug crystals start melting evidently due to the conduction mediated heat transfer from the molten polymer, however, due to the size of the drug it does not diffuse into the molten polymer or dissolve completely. The reason for maintaining the feedstock at 150 °C for one minute was that the mechanism of heating in SLS systems is radiation where the source directly interacts with the particles, whereas in HSM the heat transfer occurs through conduction (heating coil to a glass slide to the material) which takes longer. Secondly, the hatching spacing used for this work was 25 µm, which is close to the beam spot size for the laser. This allows sintering of the same area multiple times and thereby increases the exposure time. We are currently running experiments to determine the exact time spent by the laser at one point and the temperature increase following that, however, this information is currently beyond the scope of this manuscript. In this work, we attempted to simulate and visualize the ASD formation for which HSM was effectively used.

These observations from the HSM suggest that a larger drug particle size may require a longer time for diffusion, and dissolution, and thereby ASD formation. In simpler terms, a slower laser speed and a smaller particle size would translate to a more complete dissolution of the drug in the polymer matrix. To test this during the SLS 3D printing process we manufactured the tablets with the two feedstocks at different laser speeds including 50 mm/s, 75 mm/s, and 100 mm/s. The laser spends the most time in a particular region at 50 mm/s and the least time at 100 mm/s. If the HSM experiment truly demonstrates the series of events occurring during the 3D printing process, an increase in laser speed would lead to incomplete drug crystal dissolution for UPS. Post manufacturing, the tablets were crushed and observed under a polarized light microscope. As per the HSM observation, post 3D printing the granules in the case of PFS and the drug crystals in the case of UFS were engulfed by the molten polymer thereby forming a solid dispersion which can be seen in Figure 3. Incomplete drug particle incorporation in the molten polymer was observed in the case of 50 mm/s UFS and 75 mm/s UFS, however, no drug crystallinity was observed for these two laser speeds. Meanwhile, trace crystallinity was observed for 100 mm/s UFS along with incomplete drug particle incorporation. Contrary to the UFS based tablets, all the PFS-based tablets were observed to form solid dispersions without any crystallinity or with complete particle incorporation as seen in Figure 3. These observations and results suggest that the HSM can simulate the mass transfer phenomenon involved during SLS 3D printing.

### 3.2. Tablet Morphology and Quality

The tablets manufactured by SLS 3D printing using PFS and UFS were characterized for their quality attributes including physical morphology, weight, dimensions, disintegration time, and hardness. It can be seen in Table 1 that the dimensions of the tablets at all laser speeds were consistent for both UFS and PFS. Even though the dimensions and volume of the tablets were consistent, the weight of the tablets varied for different laser speeds.

There was a trend observed between the laser speed and the tablet weight where a lower laser speed led to a higher weight and vice-versa. The reason for this was the difference in the density of the tablets and the observations are consistent with the previously conducted research [39,43]. It should be noted that the density and porosity of the dosage forms impact the surface area and contribute to the performance of the tablets. This can be seen by the correlation between the disintegration time which reduces with a reduction in density (i.e., increase in porosity).

This difference in disintegration time is in seconds and can be attributed to the macro-porosity of the tablets as SLS printed dosage forms exhibit high porosity in general. Therefore, it is safe to assume that the disintegration time of the dosage form does not significantly impact the performance of the dosage form for two reasons. Firstly, all the tablets disintegrate within a couple of minutes of being exposed to the dissolution media, and secondly, the disintegration happens in the acidic pH where the drug demonstrates a very poor solubility. The difference in the laser speed may result in microporous structures in the printed tablets, which do not disintegrate rapidly and dictate the performance of the tablets. However, the presence of microporous structures and their influence on the performance of the dosage forms were not studied here.

Due to the difference in the weights of the tablets and hence the drug content, these tablets were not used for the dissolution test. Using the volume and the density of the tablets, the dimensions of the tablets with different laser speeds having the same weight and drug content were manufactured. A similar trend was observed for the hardness of the tablets where a lower laser speed resulted in higher hardness and longer disintegration time, whereas higher laser speeds reduced the hardness and led to a faster disintegration of the tablets. These findings related to the hardness and weight of the tablets were consistent for both feedstocks. Hardness correlates with disintegration time as per previous research and tablets at lower laser speeds disintegrated slower when compared to tablets manufactured at higher laser speeds. In general, the hardness, density, and disintegration for the PFS were comparatively higher when compared to UFS. The drug content of the tablets was within 90–110% of the expected drug content. The average drug content of the tablets was found to be 46.67 mg, 34.93 mg, 30.28 mg, 46.19 mg, 33.43 mg, and 27.97 mg for 50 mm/s PFS, 75 mm/s PFS, 100 mm/s PFS, 50 mm/s UFS, 75 mm/s UFS, and 100 mm/s UFS, respectively. The physical morphology and appearance of the 3D printed tablets can be seen in Figure 4. The tablets manufactured for the dissolution test with different volumes had an average drug content of 31.46 ± 2.47 mg amongst all the laser speeds and feedstocks. The drug release (%) of the tablets was calculated based on this observed average drug content.

### 3.3. Differential Scanning Calorimetry and X-ray Diffraction

Results from polarized light microscopy depicted the presence of trace crystallinity at higher laser speeds. The crushed manufactured tablet was exposed to DSC and PXRD analysis. Amorphous domains of indomethacin depict a Tg at 42 °C, which is followed by a recrystallization event to α form which is close to 100 °C as per our previously published data where we used modulated DSC [40]. For this study, we did not observe any indomethacin melting peaks which may suggest the absence of pure amorphous domains and formation of a single solution except for 100 mm/s UFS tablets which depicted recrystallization as seen in Figure 5. From the previous study, the Tg of the formulation was found to be 91 °C and that of the polymer was 120 °C. The presence of this single glass transition temperature suggests miscibility of the drug and the polymer as per Gordon-Taylor theory [40]. The DSC analysis depicted that the drug was present in its crystalline form in the UFS and had a melting point of 153 °C whereas the drug in the PFS was amorphous as it did not have a melting endotherm.

Moreover, indomethacin in all the manufactured tablets was found to be amorphous except for UFS 100 mm/s tablets. This further demonstrates and confirms the influence of the drug crystal size on ASD formation. The crystalline peak for indomethacin was small, this is due to the low drug content in the tablets, and thereby to further confirm the solid-state of the drug in the samples PXRD analysis was conducted (Figure 6). XRD confirmed the presence of crystalline peaks in 100 mm/s UFS, moreover, the XRD analysis of 75 mm/s UPS also depicted trace crystallinity which was not noticed during the DSC analysis, but was observed under the HSM.

The observations from the DSC and PXRD analysis confirm the impact of drug particle size on ASD formation during SLS 3D printing. These results also depict the role of laser speed in the manufacturing of ASD.

### 3.4. Dynamic Vapor Sorption

**Figure 7:**
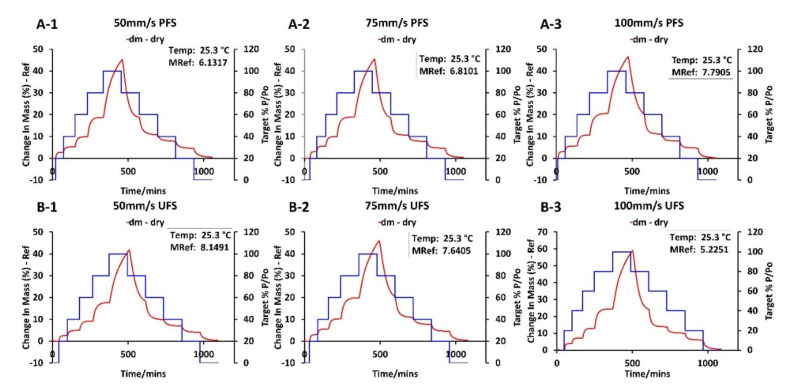
Dynamic Vapor Sorption of SLS 3D printed tablets from PFS and UFS (**A-1**) 50 mm/s PFS, (**A-2**) 75 mm/s PFS, (**A-3**) 100 mm/s PFS, (**B-1**) 50 mm/s UFS, (**B-2**) 75 mm/s UFS, (**B-3**) 100 mm/s UFS.

To inspect the stability of the SLS 3D printed tablets the crushed tablets were exposed to DVS analysis. As the step change in the %RH occurred once the weight change in the samples was stabilized, complete absorption and desorption at each %RH was ensured. The DVS analysis showed that all the samples exhibited a similar absorption and desorption trend, except for 100 mm/s UFS samples which demonstrated a higher percent weight gain as compared to the other samples (Figure 7). This higher weight gain can be attributed to the pure amorphous drug formed during the SLS 3D printing process. Previous DVS analyses have demonstrated that amorphous drugs observe a higher percent weight gain as compared to their crystalline counterpart due to high chemical potential. However, when the drug is dispersed in the polymeric matrix the amorphous drug is stabilized by the polymer, and the percent weight gain is attributed to the polymer characteristics. Furthermore, indomethacin is a good glass former and hence none of the formulations observed any recrystallization during the DVS analysis. All the samples observed maximum weight gain over 60% RH. These results indicate the stability of indomethacin ASDs over a range of humidity conditions.

### 3.5. In-Vitro Drug Release Performance Testing

The in vitro drug release performance of the manufactured tablets was investigated using non-sink, pH shift dissolution testing. Indomethacin had no solubility in the acidic pH and only released 1% drug in the non-sink conditions, whereas the pure crystalline drug release was below the limit of detection. After one hour the pH of the solution was shifted to 6.8 whilst maintaining perfect non-sink conditions. The pure drug observed a maximum solubility of 8 µg/mL. Samples were withdrawn within one minute of the pH shift where the average solubilities for 50 mm/s PFS, 75 mm/s PFS, and 100 mm/s PFS were found to be 124.54 µg/mL, 69.78 µg/mL, and 52.16 µg/mL, respectively. The solubility of indomethacin for 50 mm/s PFS, 75 mm/s PFS, and 100 mm/s PFS stabilized within 30 min of pH shift and were found to be 150 µg/mL, 90 µg/mL, and 82 µg/mL, respectively. The tablets manufactured at lower laser speeds demonstrate the highest solubility advantage (18-fold), whereas tablets at 75 mm/s and 100 mm/s demonstrate a comparatively lower solubility advantage (12-fold and 10-fold respectively). This provides further insights on the influence of the processing parameters on SLS 3D printing of ASD and further bolsters the proposed mechanism of ASD formation which is based on diffusion and dissolution of the drug in the polymeric matrix. HSM confirms the efficient diffusion of DosePlus granules in the molten polymeric matrix, however, the dissolution of the drug is dependent on the time and applied energy during the printing process. This performance test suggests that even though PFS feedstock successfully formed ASD at all the laser speeds, the dissolution, and mixing of the drug in the polymeric matrix was maximum for the formulation processed at 50 mm/s. The UFS tablets printed at 50 mm/s, 75 mm/s and 100 mm/s depicted average solubilities of 89 µg/mL, 88 µg/mL, and 51 µg/mL after one minute of pH shift and stabilized at 110 µg/mL (13-fold), 100 µg/mL (12-fold) and 60 µg/mL (7.5-fold) respectively.

**Figure 8:**
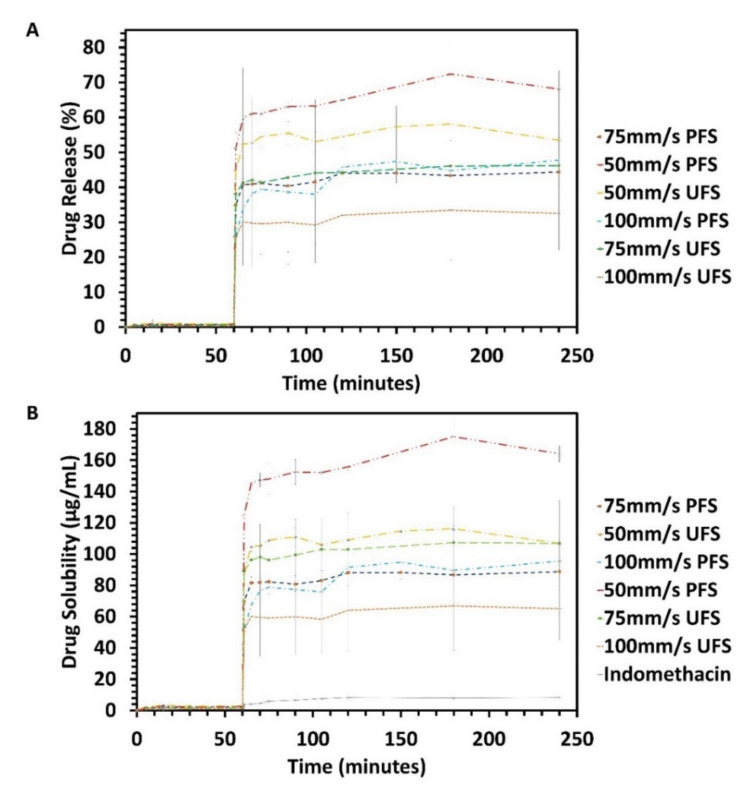
Non-sink, pH-shift in vitro performance testing of tablets printed at different laser speeds with PFS and UFS (**A**) Per-centage release of indomethacin from the 3D printed tablets (**B**) Solubility of indomethacin with different tablets.

The trend was still observed for UFS 3D printed tablets, but the solubility of 100 mm/s UFS was significantly less compared to the other printed tablets as seen in Figure 8. All tablets maintained a supersaturation throughout the dissolution test. The MANOVA analysis depicted that the laser speed significantly impacted the solubility of the drug both between (F(2,12) = 54.56, *p* < 0.0001) and within (Wilks’ Lambda = 5.83 × 10^−6^, F(18,8) = 183.65, *p* < 0.0001) the groups. The particle size of the drug (UFS and PFS) also significantly impacted the solubility of indomethacin both between (F(1,12) = 10.41, *p* = 0.0073) and within (F(9,4) = 384.85, *p* < 0.0001) the groups. Moreover, the full factorial macro also found that the combined effect of laser speed and drug particle size also had a significant effect within (F(18,8) = 122.29, *p* < 0.0001) and between (F(2,12) = 14.64, *p* = 0.0006) the groups on the solubility of indomethacin. These statistics depicted that the different drug particle sizes and laser speeds significantly influence the drug performance, and the subjects within groups demonstrate significant differences. To further elucidate which formulations are significantly different, these formulations were compared using statistical tools. MANOVA depicted that all the formulations were significantly different from one another (F(5,12) = 29.76, *p* < 0.0001). Furthermore, ANOVA analysis at T = 240 min depicted that the formulations were significantly different (F(5,12) = 26.79, *p* < 0.001) from one another, however, this is misleading as not all formulations are significantly different from each other. Comparisons of all the pairs were conducted using Tuckey-Kramer HSD, which demonstrated that the solubility of 50 mm/s PFS was significantly (*p* < 0.05) different from all the other formulations. Moreover, all the formulations were significantly (*p* < 0.05) different from 100 mm/s UFS. Apart from these pairs, the rest of the formulations were not significantly different from each other.

To inspect the state of the drug in the dissolution media and understand the mechanism of sustained supersaturation the dissolution test was repeated for 100 mm/s PFS and UFS tablets, and samples were withdrawn after 1 h in pH 2 and after 3 h in pH 6.8.

**Figure 9:**
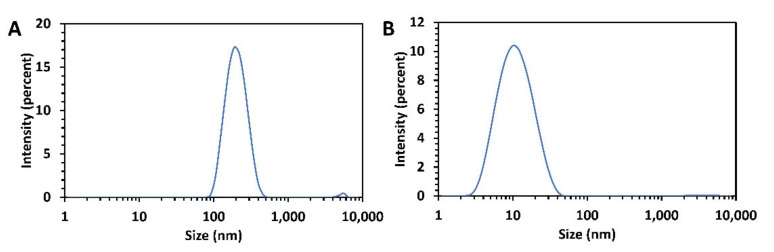
Particle size analysis for the PFS 100 mm/s tablets at pH 2 (**A**) Unfiltered sample (**B**) Filtered sample (0.2 µm PES filter).

The particle size of the unfiltered dissolution media and filtered dissolution media were determined. It was determined that the particles in acidic pH were evenly dispersed around 200 nm for PFS 100 mm/s samples and unevenly polydisperse for 100 mm/s UFS samples. Furthermore, these evenly dispersed 100 mm/s PFS samples were filtered and analyzed, wherein uniformly dispersed nanoparticles in the range of 9 nm were detected. Nanoparticles were also detected in the 100 mm/s UFS samples; however, the samples were unevenly polydisperse over 200 nm in unfiltered samples and less than 200 nm in the filtered samples. In a nutshell, the nanoparticles in the UFS and PFS samples had similar mean particle sizes, but the latter had a lower PDI as seen in Table 2 and Figure 9.

These nanoparticles explain the maintained supersaturation even at non-sink conditions and complement the observations made by Jara et al. (2021) where ASD of niclosamide was manufactured using Kollidon^®^ VA 64 as a carrier [44,45]. This also suggests that the determined solubility was the apparent solubility and not the true solubility of indomethacin. These polymer-stabilized nanoparticles maintain the apparent solubility of indomethacin in non-sink conditions and may contribute to in vivo bioavailability enhancement in sink conditions. Moving forward it was critical to understand the nature of these nanoparticles i.e., whether these particles were crystalline or amorphous. However, filtration and drying of the particle might stimulate crystallization. Hence, to determine the nature of the nanoparticles without filtration and drying, the samples were collected in the same manner as described for the particle size analysis and analyzed using WAXS.

**Figure 10:**
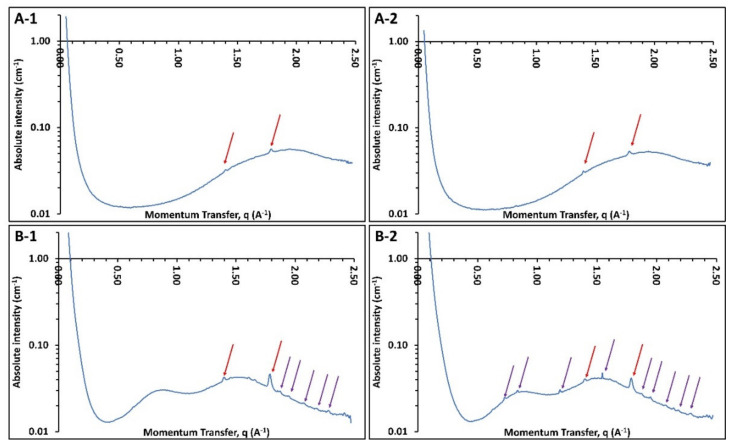
Wide-angle X-ray diffraction of samples withdrawn from the dissolution vessels, (**A-1**) 100 mm/s PFS at pH 2, (**A-2**) 100 mm/s PFS at pH 6.8, (**B-1**) 100 mm/s UFS at pH 2, (**B-2**) 100 mm/s UFS at pH 6.8.

Figure 10 depicts the nature of the suspended particles in the dissolution medium. It is apparent that the samples from 100 mm/s PFS tablets do not show any indomethacin crystalline peaks before or after the pH shift and the only visible peaks (red arrows) belong to the sintering agent (Candurin^®^). Meanwhile, the 100 mm/s UFS samples demonstrate small crystalline peaks at pH 2 which might be due to the trace crystallinity in the 100 mm/s UFS samples as seen from the XRD analysis. However, 100 mm/s UFS samples demonstrate an increase in the crystalline peak intensity post pH shift. This increase in crystalline peaks may be an indicator of recrystallization in the dissolution medium and the potential instability of the formulation [46]. Indomethacin is a good glass former and hence is relatively stable as compared to drugs with the poor glass-forming ability [31,47,48,49]. However, trace crystallinity in ASD can lead to not only storage instability but also solution instability leading to unexpected performance of the dosage forms [50].

## 4. Discussion

3D printing of amorphous solid dispersions with improved solubility and dissolution is a relatively new application of selective laser sintering. The study published by Davis et al. demonstrated the application of selective laser sintering for the manufacturing of ritonavir-Kollidon^®^ VA 64 ASDs with a 21-fold increase in its solubility. In that study, it was observed that the laser speed and the hatch spacing were critical for manufacturing amorphous solid dispersion [25]. Furthermore, these ASDs were compared with hot-melt extruded solid-dispersions using different solid-state characterizations, where the solid-state NMR and in vitro performance depicted the similarity between these two ASDs manufactured by different techniques [25]. However, it was observed that the drug’s poor flow properties negatively affect the SLS process and the uniformity of the dosage forms. Previous studies on SLS 3D printing have stressed the importance of flow properties of the feedstock and their impact on the physical properties of the printed part [51]. A study by Brika et al. on laser powder bed fusion (LPBF) additive manufacturing of Ti-6Al-4V powders with different particle morphologies observed that the powder particles’ morphology, size, and density influence the flowability of these powders. This study concluded that the use of highly spherical powders promotes feedstock flow properties which forms printed parts with better physical and geometric characteristics [51]. To improve the flow properties of challenging drugs and thereby facilitate the 3D printing of uniform dosage forms, in our last study we developed a hot melt extrusion-based granulation platform, where the drug is processed with spherical inorganic excipients, and the drug is absorbed onto the excipient surface [40]. This platform successfully 3D-printed amorphous solid dispersions with uniform and reproducible dosage forms. From the previous studies, it was evident that the hatch spacing and laser speed, along with formulation flow properties play a critical role in 3D printing solid dispersions, however, the mechanism of ASD formation using SLS has not been evaluated previously. In this study, we prepared two feedstocks with different drug particle sizes where the crystalline drug’s size was over 50 µm whereas the drug absorbed on the inorganic carrier was below 5 µm. The ASD formation during an SLS process was simulated using HSM. We observed that the drug interacts with the molten polymer by diffusion, and dissolves in the molten polymer by means of dissolution. It was also seen that smaller drug particle size facilitated dissolution in the polymer, whereas larger drug particle size led to incomplete drug dissolution. These results were confirmed by observing the SLS tablets using a polarized light microscope where the trace crystallinity and incomplete drug dissolution were observed for larger drug particle sizes. These observations align with previous studies conducted by Nele-Johanna et al., where crystalline celecoxib with two different particle sizes was dispersed into polyvinyl pyrrolidone using microwave irradiation [52]. It was observed that the celecoxib with a smaller particle size (<71 µm) observed a faster and more complete amorphous conversion as compared to the drug crystals with a particle size over 71 µm [52].

The energy density on a certain region induced by the laser correlates with hatch spacing and laser speed set for the process [39,53]. A smaller hatch spacing and slower laser speeds lead to an increase in the time spent by the laser on the exposed region. The influence of laser speed was seen on the formation of ASDs in this study, where slower laser speeds formed ASDs with better performance as the laser spends more time on the feedstock, leading to a more complete dissolution of the drug in the polymer matrix, in contrast to a faster laser speed where the laser spends less time on a certain region. This can be observed in the PLM images where tablets manufactured at faster laser speeds observed incomplete dissolution of the drug in the polymer matrix for the feedstock with the drug having a larger particle size. These observations from the microscopy were confirmed by DSC and XRD results, where the tablets manufactured at 100 mm/s for UFS observed trace crystallinity, moreover, this formulation also observed the presence of amorphous content from the DVS analysis. A recent study conducted by Dana et al. demonstrated that the trace or residual crystallinity in bicalutamide (BCL)/polyvinylpyrrolidone vinyl acetate copolymer (PVPVA) ASDs may negatively influence the performance by reducing the solubility advantage due to seeding crystal growth which can, in turn, lead to the de-supersaturation [50]. This trace crystallinity also negatively influenced the release and solubility of the SLS 3D printed ASDs where the tablets manufactured using UFS at 100 mm/s observed a significantly lower solubility as compared to the rest of the formulations, whereas the tablets manufactured at 50 mm/s using PFS observed a significantly higher solubility and release as compared to the rest of the formulations. All the other formulations observed a trend where slower laser speeds and smaller drug particle sizes depicted a better performance and faster laser speeds with larger drug particle sizes depicted a comparatively inferior performance. Both these parameters i.e., drug particle size and laser speed significantly influenced the solubility of the drug which further strengthens the proposed mechanism of ASD formation using SLS. Unlike Dana et al., de-supersaturation of the formulations with residual crystallinity was not observed which can be attributed to the good glass-forming ability of indomethacin [48,54]. However, this de-supersaturation phenomenon might be observed in non-sink conditions at faster laser speeds for larger drug particle sizes in the cases where poor glass-forming drugs (rapid crystallizers) are processed with SLS 3D printing.

## 5. Conclusions

This study demonstrated the mechanism and the impact of drug particle size on amorphous solid dispersion (ASD) formation by selective laser sintering (SLS) 3D printing. It is apparent from the conditions simulated using hot-stage microscopy that the mechanism of ASD formation is the diffusion and dissolution of the drug in the molten polymeric matrix. We further demonstrated that the drug particle size plays a critical role in the diffusion and dissolution of the drug where a lower drug particle size (<5 µm) leads to a faster dissolution and intense mixing of the drug and the polymer in comparison to drug crystals with higher particle size (>50 µm). Furthermore, feedstock containing processed granules can form stable ASDs even at 100 mm/s laser speed whereas unprocessed drugs retain trace crystallinity and exhibit a weak supersaturation and instability under the same processing conditions. This study concludes that processed granules can expand the processing design space for ASD manufacturing using SLS 3D printing.

## Figures and Tables

**Table 1:** Quality attributes of 3D printed tablets using PFS and UFS.

Tablet Batch No.	Height (mm)	Diameter (mm)	Weight (mg)	Volume (mm^3^)	Density (kg/m^3^)	Hardness (kp)	Disintegration Time (s)
50 mm/s PFS	6.57 ± 0.06	12.90 ± 0.26	471 ± 4.04	857.82	0.549	8.66 ± 0.24	67 ± 5
75 mm/s PFS	6.43 ± 0.06	12.77 ± 0.12	367 ± 8.74	823.12	0.446	6.35 ± 0.17	45 ± 12
100 mm/s PFS	6.37 ± 0.06	12.97 ± 0.15	306 ± 6.56	840.31	0.364	4.56 ± 0.12	25 ± 9
50 mm/s UFS	6.53 ± 0.15	12.87 ± 0.15	457 ± 10.41	849.06	0.538	7.52 ± 0.39	58 ± 14
75 mm/s UFS	6.43 ± 0.06	12.53 ± 0.47	346 ± 6.56	793.30	0.436	6.03 ± 0.19	36 ± 8
100 mm/s UFS	6.23 ± 0.06	12.80 ± 0.10	287 ± 6.56	801.70	0.358	4.00 ± 0.33	7 ± 3

**Table 2:** Mean particle size distribution in filtered and unfiltered dissolution medium.

Sample	Mean Particle Size	PDI
Unfiltered 100 mm/s UFS	206.20 ± 9.95	0.399 ± 0.087
Filtered 100 mm/s UFS	9.87 ± 0.03	0.219 ± 0.001
Unfiltered 100 mm/s PFS	200.83 ± 1.12	0.272 ± 0.030
Filtered 100 mm/s PFS	9.61 ± 0.02	0.200 ± 0.003

## Supplementary Materials

The following are available online at https://www.mdpi.com/article/10.3390/pharmaceutics13081149/s1, Figure S1: Particle size distribution (PSD) of (A) unprocessed (UFS) and (B) processed feedstock, Table S1: Particle size distribution of sieved UFS and PFS for SLS.

## References

[1] Awad A., Fina F., Goyanes A., Gaisford S., Basit A.W. (2020). 3D printing: Principles and pharmaceutical applications of selective laser sintering. Int. J. Pharm..

[2] Wang J., Zhang Y., Aghda N.H., Pillai A.R., Thakkar R., Nokhodchi A., Maniruzzaman M. (2021). Emerging 3D printing technologies for drug delivery devices: Current status and future perspective. Adv. Drug Deliv. Rev..

[3] Aho J., Bøtker J.P., Genina N., Edinger M., Arnfast L., Rantanen J. (2019). Roadmap to 3D-Printed Oral Pharmaceutical Dosage Forms: Feedstock Filament Properties and Characterization for Fused Deposition Modeling. J. Pharm. Sci..

[4] Cheng Y., Qin H., Acevedo N.C., Shi X. (2021). Development of methylcellulose-based sustained-release dosage by semisolid extrusion additive manufacturing in drug delivery system. J. Biomed. Mater. Res. Part B Appl. Biomater..

[5] Conceição J., Vaamonde X.F., Goyanes A., Adeoye O., Concheiro A., Cabral-Marques H., Lobo J.M.S., Alvarez-Lorenzo C. (2019). Hydroxypropyl-β-cyclodextrin-based fast dissolving carbamazepine printlets prepared by semisolid extrusion 3D printing. Carbohydr. Polym..

[6] Ziaee M., Crane N.B. (2019). Binder jetting: A review of process, materials, and methods. Addit. Manuf..

[7] Zhang Y., Zhang J., Thakkar R., Pillai A.R., Wang J., Lu A., Maniruzzaman M. (2021). Functions of Magnetic Nanoparticles in Selective Laser Sintering (SLS) 3D Printing of Pharmaceutical Dosage Forms. ChemRxiv.

[8] Kruth J., Mercelis P., Van Vaerenbergh J., Froyen L., Rombouts M. (2005). Binding mechanisms in selective laser sintering and selective laser melting. Rapid Prototyp. J..

[9] Bai Y., Wall C., Pham H., Esker A., Williams C. (2018). Characterizing Binder–Powder Interaction in Binder Jetting Additive Manufacturing Via Sessile Drop Goniometry. J. Manuf. Sci. Eng..

[10] González G., Baruffaldi D., Martinengo C., Angelini A., Chiappone A., Roppolo I., Pirri C., Frascella F. (2020). Materials Testing for the Development of Biocompatible Devices through Vat-Polymerization 3D Printing. Nanomaterials.

[11] Martinez P.R., Goyanes A., Basit A.W., Gaisford S. (2017). Fabrication of drug-loaded hydrogels with stereolithographic 3D printing. Int. J. Pharm..

[12] Zeng K., Pal D., Stucker B., A Review of Thermal Analysis Methods in Laser Sintering and Selective Laser Melting 23rd Annual International Solid Freeform Fabrication Symposium—An Additive Manufacturing Conference, SFF 2012. http://utw10945.utweb.utexas.edu/Manuscripts/2012/2012-60-Zeng.pdf.

[13] Allahham N., Fina F., Marcuta C., Kraschew L., Mohr W., Gaisford S., Basit A.W., Goyanes A. (2020). Selective Laser Sintering 3D Printing of Orally Disintegrating Printlets Containing Ondansetron. Pharmaceutics.

[14] Fina F., Goyanes A., Gaisford S., Basit A.W. (2017). Selective laser sintering (SLS) 3D printing of medicines. Int. J. Pharm..

[15] Fina F., Madla C.M., Goyanes A., Zhang J., Gaisford S., Basit A.W. (2018). Fabricating 3D printed orally disintegrating printlets using selective laser sintering. Int. J. Pharm..

[16] Leong K.F., Chua C.K., Gui W.S. (2006). Verani Building Porous Biopolymeric Microstructures for Controlled Drug Delivery Devices Using Selective Laser Sintering. Int. J. Adv. Manuf. Technol..

[17] Salmoria G.V., Klauss P., Kanis L.A. (2017). Laser Printing of PCL/Progesterone Tablets for Drug Delivery Applications in Hormone Cancer Therapy. Lasers Manuf. Mater. Process..

[18] Salmoria G., Klauss P., Zepon K.M., Kanis L., Roesler C., Vieira L. (2012). Development of functionally-graded reservoir of PCL/PG by selective laser sintering for drug delivery devices. Virtual Phys. Prototyp..

[19] Awad A., Fina F., Trenfield S.J., Patel P., Goyanes A., Gaisford S., Basit A.W. (2019). 3D Printed Pellets (Miniprintlets): A Novel, Multi-Drug, Controlled Release Platform Technology. Pharmaceutics.

[20] Fina F., Goyanes A., Madla C.M., Awad A., Trenfield S.J., Kuek J.M., Patel P., Gaisford S., Basit A.W. (2018). 3D printing of drug-loaded gyroid lattices using selective laser sintering. Int. J. Pharm..

[21] Salmoria G.V., Vieira F.E., Ghizoni G.B., Gindri I.M., Kanis L.A. (2017). Additive Manufacturing of PE/Fluorouracil Waffles for Implantable Drug Delivery in Bone Cancer Treatment. Int. J. Eng. Res. Sci..

[22] Salmoria G., Cardenuto M., Roesler C., Zepon K., Kanis L. (2016). PCL/Ibuprofen Implants Fabricated by Selective Laser Sintering for Orbital Repair. Procedia CIRP.

[23] Gv S., Fe V., Gb G., Ms M., La K. (2017). 3D printing of PCL/Fluorouracil tablets by selective laser sintering: Properties of implantable drug delivery for cartilage cancer treatment. Rheumatol. Orthop. Med..

[24] Salmoria G., Vieira F., Muenz E., Gindri I., Marques M., Kanis L. (2018). Additive Manufacturing of PE/fluorouracil/progesterone intrauterine device for endometrial and ovarian cancer treatments. Polym. Test..

[25] Davis D.A., Thakkar R., Su Y., Williams R.O., Maniruzzaman M. (2021). Selective Laser Sintering 3-Dimensional Printing as a Single Step Process to Prepare Amorphous Solid Dispersion Dosage Forms for Improved Solubility and Dissolution Rate. J. Pharm. Sci..

[26] Abramov Y.A., Sun G., Zeng Q., Zeng Q., Yang M. (2020). Guiding Lead Optimization for Solubility Improvement with Physics-Based Modeling. Mol. Pharm..

[27] Lipp R. (2013). The innovator pipeline: Bioavailability challenges and advanced oral drug delivery opportunities. Am. Pharm. Rev..

[28] Brouwers J., Brewster M.E., Augustijns P. (2009). Supersaturating Drug Delivery Systems: The Answer to Solubility-Limited Oral Bioavailability?. J. Pharm. Sci..

[29] Augustijns P., Brewster M.E. (2012). Supersaturating Drug Delivery Systems: Fast is Not Necessarily Good Enough. J. Pharm. Sci..

[30] Pandi P., Bulusu R., Kommineni N., Khan W., Singh M. (2020). Amorphous solid dispersions: An update for preparation, characterization, mechanism on bioavailability, stability, regulatory considerations and marketed products. Int. J. Pharm..

[31] Wyttenbach N., Kuentz M. (2017). Glass-forming ability of compounds in marketed amorphous drug products. Eur. J. Pharm. Biopharm..

[32] DiNunzio J.C., Brough C., Miller D.A., Williams R.O., McGINITY J.W. (2010). Applications of KinetiSol^®^ Dispersing for the production of plasticizer free amorphous solid dispersions. Eur. J. Pharm. Sci..

[33] Jermain S.V., Lowinger M.B., Ellenberger D.J., Miller D.A., Su Y., Williams I.R.O., Iii R.W. (2020). In Vitro and In Vivo Behaviors of KinetiSol and Spray-Dried Amorphous Solid Dispersions of a Weakly Basic Drug and Ionic Polymer. Mol. Pharm..

[34] Jermain S.V., Miller D., Spangenberg A., Lu X., Moon C., Su Y., Williams R.O. (2019). Homogeneity of amorphous solid dispersions—An example with KinetiSol^®^. Drug Dev. Ind. Pharm..

[35] DiNunzio J.C., Brough C., Hughey J.R., Miller D.A., Iii R.W., McGINITY J.W. (2010). Fusion production of solid dispersions containing a heat-sensitive active ingredient by hot melt extrusion and Kinetisol^®^ dispersing. Eur. J. Pharm. Biopharm..

[36] Gala U., Miller D., Iii R.O.W. (2020). Improved Dissolution and Pharmacokinetics of Abiraterone through KinetiSol^®^ Enabled Amorphous Solid Dispersions. Pharmaceutics.

[37] Hughey J.R., DiNunzio J.C., Bennett R.C., Brough C., Miller D.A., Ma H., Iii R.W., McGINITY J.W. (2010). Dissolution Enhancement of a Drug Exhibiting Thermal and Acidic Decomposition Characteristics by Fusion Processing: A Comparative Study of Hot Melt Extrusion and KinetiSol^®^ Dispersing. AAPS Pharm. Sci. Tech..

[38] Thakkar R., Thakkar R., Pillai A., Ashour E.A., Repka M.A. (2020). Systematic screening of pharmaceutical polymers for hot melt extrusion processing: A comprehensive review. Int. J. Pharm..

[39] Thakkar R., Davis D.A., Williams R.O., Maniruzzaman M. (2021). Selective Laser Sintering of a Photosensitive Drug: Impact of Processing and Formulation Parameters on Degradation, Solid-State, and Quality of 3D Printed Dosage Forms. bioRxiv.

[40] Thakkar R., Zhang Y., Zhang J., Maniruzzaman M. (2021). Synergistic application of twin-screw granulation and selective laser sintering 3D printing for the development of pharmaceutical dosage forms with enhanced dissolution rates and physical properties. Eur. J. Pharm. Biopharm..

[41] Thakkar R., Pillai A.R., Zhang J., Zhang Y., Kulkarni V., Maniruzzaman M. (2020). Novel On-Demand 3-Dimensional (3-D) Printed Tablets Using Fill Density as an Effective Release-Controlling Tool. Polymers.

[42] Sun D.D., Wen H., Taylor L.S. (2016). Non-Sink Dissolution Conditions for Predicting Product Quality and In Vivo Performance of Supersaturating Drug Delivery Systems. J. Pharm. Sci..

[43] Ali S.F.B., Mohamed E., Ozkan T., Kuttolamadom M., Khan M.A., Asadi A., Rahman Z. (2019). Understanding the effects of formulation and process variables on the printlets quality manufactured by selective laser sintering 3D printing. Int. J. Pharm..

[44] Jara M., Warnken Z., Williams R. (2021). Amorphous Solid Dispersions and the Contribution of Nanoparticles to In Vitro Dissolution and In Vivo Testing: Niclosamide as a Case Study. Pharmaceutics.

[45] Ilevbare G.A., Taylor L.S. (2013). Liquid–Liquid Phase Separation in Highly Supersaturated Aqueous Solutions of Poorly Water-Soluble Drugs: Implications for Solubility Enhancing Formulations. Cryst. Growth Des..

[46] Luebbert C., Sadowski G. (2017). Moisture-induced phase separation and recrystallization in amorphous solid dispersions. Int. J. Pharm..

[47] Blaabjerg L.I., Lindenberg E., Löbmann K., Grohganz H., Rades T. (2018). Is there a correlation between the glass forming ability of a drug and its supersaturation propensity?. Int. J. Pharm..

[48] Blaabjerg L.I., Bulduk B., Lindenberg E., Löbmann K., Rades T., Grohganz H. (2019). Influence of Glass Forming Ability on the Physical Stability of Supersaturated Amorphous Solid Dispersions. J. Pharm. Sci..

[49] Baird J.A., Van Eerdenbrugh B., Taylor L. (2010). A Classification System to Assess the Crystallization Tendency of Organic Molecules from Undercooled Melts. J. Pharm. Sci..

[50] Moseson D.E., Corum I.D., Lust A., Altman K.J., Hiew T.N., Eren A., Nagy Z.K., Taylor L.S. (2021). Amorphous Solid Dispersions Containing Residual Crystallinity: Competition between Dissolution and Matrix Crystallization. AAPS J..

[51] Brika S.E., Letenneur M., Dion C.A., Brailovski V. (2020). Influence of particle morphology and size distribution on the powder flowability and laser powder bed fusion manufacturability of Ti-6Al-4V alloy. Addit. Manuf..

[52] Hempel N., Knopp M.M., Berthelsen R., Zeitler J.A., Löbmann K. (2020). The influence of drug and polymer particle size on the in situ amorphization using microwave irradiation. Eur. J. Pharm. Biopharm..

[53] Yap C.Y., Chua C.K., Dong Z.L., Liu Z.H., Zhang D.Q., Loh L.E., Sing S.L. (2015). Review of selective laser melting: Materials and applications. Appl. Phys. Rev..

[54] Blaabjerg L.I., Lindenberg E., Löbmann K., Grohganz H., Rades T. (2016). Glass Forming Ability of Amorphous Drugs Investigated by Continuous Cooling and Isothermal Transformation. Mol. Pharm..

