# Supplementary material for: Impact of Laser Speed and Drug Particle Size on Selective Laser Sintering 3D Printing of Amorphous Solid Dispersions
Source: Pharmaceutics. 2021 Jul 27;13(8):1149. doi: 10.3390/pharmaceutics13081149 (PMC8400191; doi:10.3390/pharmaceutics13081149)
Supplement: Supplementary file 1 [file pharmaceutics-13-01149-s001.zip › pharmaceutics-1290076-supplementary.pdf]

pharmaceutics

Supplementary Material: Impact of Laser Speed and Drug Particle Size on Selective Laser Sintering 3D Printing of Amorphous Solid Dispersions

Rishi Thakkar, Miguel O. Jara, Steve Swinnea, Amit R. Pillai and Mohammed Maniruzzaman

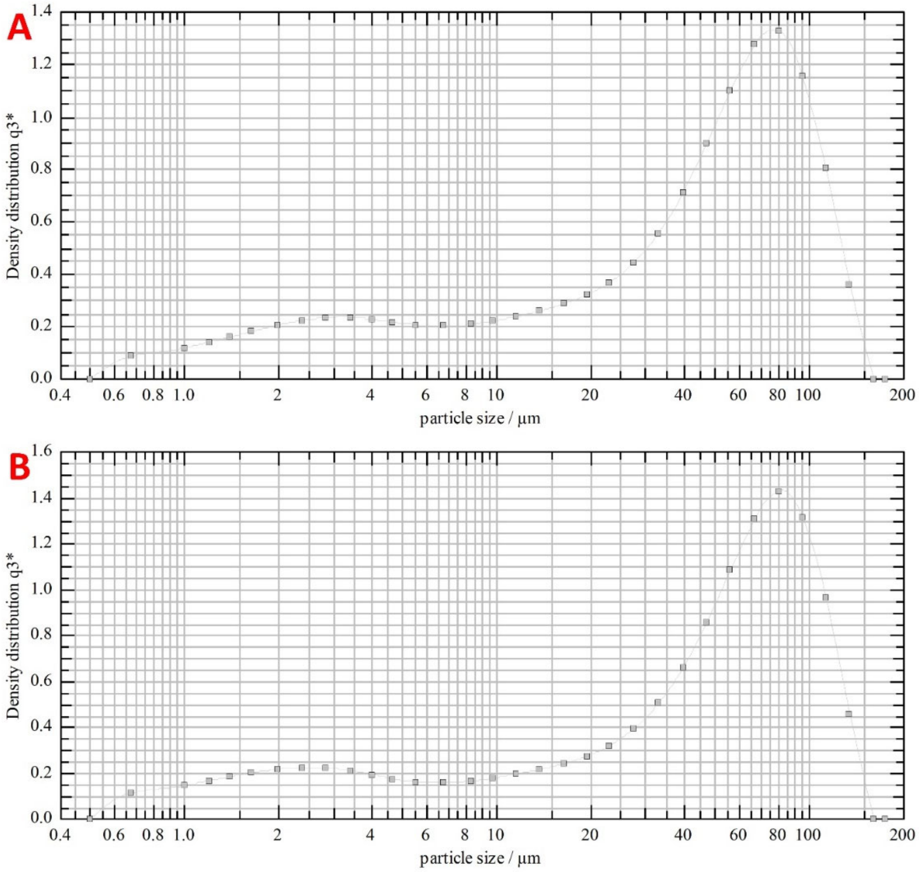

Figure S1. Particle size distribution (PSD) of A) unprocessed (UFS) and B) processed feedstock.

Table S1. Particle size distribution of sieved UFS and PFS for SLS.

Material	d_{10} (µm)	d_{50} (µm)	d_{90} (µm)	Span (µm)
**UFS**	2.63	46.15	100.96	98.38
**PFS**	2.28	50.99	105.47	103.19
